# Multiscale Entropy Analysis of Postural Stability for Estimating Fall Risk via Domain Knowledge of Timed-Up-And-Go Accelerometer Data for Elderly People Living in a Community

**DOI:** 10.3390/e21111076

**Published:** 2019-11-02

**Authors:** Chi-Han Wu, Chia-Hsuan Lee, Bernard C. Jiang, Tien-Lung Sun

**Affiliations:** 1 Department of Industrial Engineering and Management, Yuan Ze University, 135 Yuan Tung Road, Chungli District, Taoyuan 320, Taiwan; s1038903@mail.yzu.edu.tw; 2 Department of Industrial Management, National Taiwan University of Science and Technology, No. 43, Sec. 4, Keelung Road, Da’an District, Taipei 106, Taiwan; sweat0430@mail.ntust.edu.tw (C.-H.L.); bcjiang@mail.ntust.edu.tw (B.C.J.)

**Keywords:** multiscale entropy, complexity index (CI), timed up and go (TUG), segment-based TUG (sTUG), Sit-to-Walk (STW), Walk, Turning, Walk-to-Sit (WTS)

## Abstract

As people in developed countries live longer, assessing the fall risk becomes more important. A major contributor to the risk of elderly people falling is postural instability. This study aimed to use the multiscale entropy (MSE) analysis to evaluate postural stability during a timed-up-and-go (TUG) test. This test was deemed a promising method for evaluating fall risk among the elderly in a community. The MSE analysis of postural instability can identify the elderly prone to falling, whereupon early medical rehabilitation can prevent falls. Herein, an objective approach is developed for assessing the postural stability of 85 community-dwelling elderly people (aged 76.12 ± 6.99 years) using the short-form Berg balance scale. Signals were collected from the TUG test using a triaxial accelerometer. A segment-based TUG (sTUG) test was designed, which can be obtained according to domain knowledge, including “Sit-to-Walk (STW),” “Walk,” “Turning,” and “Walk-to-Sit (WTS)” segments. Employing the complexity index (CI) of sTUG can reveal information about the physiological dynamics’ signal for postural stability assessment. Logistic regression was used to assess the fall risk based on significant features of CI related to sTUG. MSE curves for subjects at risk of falling (*n* = 19) exhibited different trends from those not at risk of falling (*n* = 66). Additionally, the CI values were lower for subjects at risk of falling than those not at risk of falling. Results show that the area under the curve for predicting fall risk among the elderly subjects with complexity index features from the overall TUG test is 0.797, which improves to 0.853 with the sTUG test. For the elderly living in a community, early assessment of the CI for sTUG using MSE can help predict the fall risk.

## 1. Introduction

Preventing falls is always an important issue in clinical preventive medicine. In particular, falls for the elderly are likely to lead to major injuries, long-term disability, reduced activity or mobility, or even death. According to a report by the World Health Organization, a person aged 65–70 years has a risk of 28%–35% of falling, increasing to 32%–42% for those aged over 70 years [1]. According to a previous study [2], 33% of the elderly living in a community have experienced a fall event, and 50% fall repeatedly. The elderly people who have either fallen or possess a gait or balance problem are at higher risk of future falls [3,4]. Moreover, correct assessment of postural stability is considered an important predictor of fall risk [5,6]. An effective fall prevention programme first identifies the elderly at risk of falling, and then determines the most appropriate interventions, thereby reducing or eliminating preventable falls.

However, the continuous monitoring of gait and postural stability requires extensive healthcare and clinical resources. Limited professional resources (e.g., physiotherapists, nurses, and doctors) relative to the growing ageing population worldwide are insufficient to detect postural instability in a timely fashion, which could, therefore, result in many falls that could have been avoided through continuous monitoring and early interventions. To fill such a gap between resources and care needs, an approach for the timely assessment of balance among the community-dwelling elderly people that does not require healthcare professionals’ involvement is urgently needed. In addition, wearable systems based on inertial sensors that are light, portable, and cheap allow body motion to be quantitatively measured [7]. Therefore, to support medical professionals for clinically assessing fall risk, the combination of (i) a wearable device; (ii) feature analysis; and (iii) statistical analysis techniques is used as an auxiliary tool.

The 3MTUG test is a widely used and accepted clinical test for assessing functional mobility [8]. It is simple and easy-to-administer anywhere and anytime. Fundamentally, 3MTUG comprises a set of basic mobility skills that are vital to independent living; thus, it has been suggested as a useful screening tool for identifying elderly with balance or gait deficits [9]. In this study, we investigate the effectiveness of the 3MTUG test by means of a waist-mounted triaxial accelerometer to estimate the short-form Berg balance scale (SFBBS) for assessing the postural stability of a community-dwelling elderly person. The SFBBS, which is psychometrically similar (including test reliability, validity, and responsiveness) to the original Berg balance scale (BBS) [10], is a widely used tool for assessing a person’s static and dynamic balance abilities and a reliable measure of balance impairment, thereby providing an effective fall-risk prediction [11].

Multiscale entropy (MSE), proposed by Costa et al., is an entropy-based measure for time series that is applicable to quantifying the ‘complexity’ of physiologic and physical signals of finite length [12,13]. MSE analysis examines not only the magnitude of the sample entropy but also patterns of responses on different time scales. Additionally, Costa et al. [14] presented an MSE algorithm that can be used to measure complexity over a range of scales and showed that normal spontaneous walking exhibits high complexity when compared to slow gait. Moreover, MSE techniques are notable because they probe a dynamic property not identified by other statistics and have implications for quantifying and modelling gait control under physiological and pathological conditions. In addition, MSE analysis has been widely used in applications such as measuring gait stability [11,14,15] and predicting diseases [16,17]. For the 3MTUG test, MSE may be a more sensitive indicator than accelerometer features, such as slope, range, and duration time, when evaluating the fall risk for community-dwelling elderly people [11]. Therefore, we used MSE to quantify the complexity of a time series on multiple spatiotemporal scales, where complexity can be related to the fall risk for the elderly.

In a previous study, MSE analysis of the overall TUG was shown to offer a good accelerometer-based measurement, which has been used to categorise the falling behaviour of the community-dwelling elderly people [11]. However, this study did not shed any light on which specific TUG aspects showed impaired postural stability. The TUG subtasks included “Sit-to-Walk (STW),” “Walk,” “Turning,” and “Walk-to-Sit (WTS)” segments; this segmentation aims to capture the different characteristics of various body movements. For example, both “Walk” and “Turning” segments are related to locomotion (e.g., reciprocal and rhythmic activation of the left and right lower legs), whereas “STW” and “WTS” segments are largely related to the strength and power of the lower extremities [18]. However, the potential mechanisms of these TUG subtasks may be different from each other. Moreover, some previous studies have referred to segments of the TUG test and extracted subtask features to perform an evaluation or estimation related to physiological risk factors predisposing one to fall risk [19,20,21]. In this study, we investigated an overall TUG test and a segment-based TUG (sTUG) test. The latter was comprised of STW, Walk, Turning and WTS segments, which were obtained after subdividing the TUG test into four segments based on the domain knowledge about the four types of activities. Therefore, we focused on the segment-based features extracted from a wearable sensor and tried to use MSE analysis to investigate their influence on postural stability for fall-risk assessment. Logistic regression was used to assess postural stability for fall risk based on significant CI features of the overall TUG and sTUG. We hypothesised that (1) the MSE of sTUG offers a more robust indicator for evaluating fall risk compared to that of the overall TUG; (2) the MSE is higher for non-fallers than fallers, and (3) the MSE values of Walk/Turning segments are higher than those of STW/WTS segments.

## 2. Materials and Methods

### 2.1. Subjects

We originally recruited 91 community-dwelling elderly people in a regional hospital in central Taiwan between April 2014 and May 2015 and formed a team of medical professionals, including rehabilitation physicians, physiotherapists, and functional therapists, to conduct a 3MTUG test to assess postural stability. Prior to the assessment, written consent was obtained from the participants. The conditions for accepting a subject were that she/he should (i) be over 65 years old; (ii) have had no musculoskeletal injuries or problems of the central nervous system in the past three months; and (iii) be able to walk independently without any aid. However, because of gaps in some of the collected signals, the actual sample size was only 85 elderly (average age: 76.12 ± 6.99 years; 18 men, average age 78.89 ± 5.95 years; 67 women, average age 75.37 ± 7.1 years). This study was approved by the Institutional Review Board of Tsaotun Psychiatric Center, Ministry of Health and Welfare’s research ethics board, on 19 May 2015 (project approval number 104013). All subjects provided written informed consent to participate in the study prior to entering the laboratory.

### 2.2. Sensor

**Figure 1:**
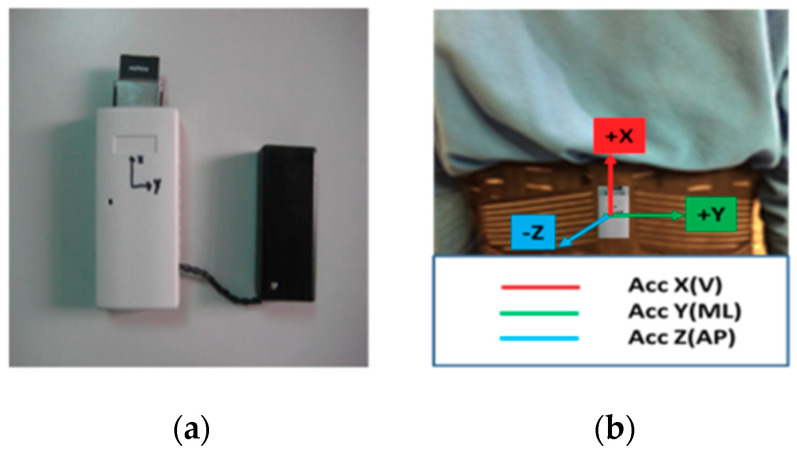
(**a**) Sensor; (**b**) placement location and corresponding axes/directions.

In this experiment, a triaxial accelerometer (RD3152MMA7260Q; Freescale Semiconductor-NXP, Astin, TX, USA; sampling rate 45 Hz) was placed on the subject’s back at vertebrae L3–L5 during the 3MTUG test (see Figure 1). This position is the centre of gravity of the human body, and most research [7] on fall-risk assessment has used this position. The X, Y, and Z axes were aligned with the vertical (V; up: +, down: −), mediolateral (ML; right: +, left: −), and anterior-posterior (AP; forward: +, backward: −) directions, respectively.

### 2.3. Clinical Test

The hospital staff, including rehabilitation doctors, physical therapists, and nurses, visited elderly community service centres in central Taiwan to advocate fall-risk prevention concepts and conduct fall-risk assessments. The SFBBS can be used for a clinical fall-risk assessment of balance. With the advancement of Internet of Things, sensors are also considered to be risk assessment tools. The present study considered the community-dwelling elderly people and performed measurements using both clinical tests and inertial sensors. The methods used for assessing the fall risk included MSE analysis, multivariable logistic regression, receiver operating characteristic (ROC), area under the ROC curve (AUC), and clinical tests. On average, each elderly person spent approximately 15–20 min completing all the assessment tests, such as SFBBS and TUG. Simultaneously, axis data were collected via a TUG test using sensors. In this study, we used the MSE of overall TUG and sTUG to perform nonlinear analysis to explore the differences between those at risk of falling and those not at risk of falling.

#### 2.3.1. SFBBS

As a simplified version of the BBS, the SFBBS is a reliable evaluation tool used to assess the balance of the elderly [11]. It is comprised of seven activities [22]; namely, (i) reaching forward with arms outstretched; (ii) standing with eyes closed; (iii) standing with one foot in front, (iv) turning to look behind; (v) retrieving an object from the floor; (vi) standing on one foot, and (vii) moving from sitting to standing. Compared to the BBS, the SFBBS requires only half the time (approximately 10–15 min) to complete all activities. The clinical expert must explain all SFBBS actions to all subjects and demonstrate details when necessary. Those specified actions are arranged in order by degree of difficulty. The clinical expert scores each subject according to the completion status. Each of the seven items is divided into three criteria: (a) unable to perform the action (zero points); (b) completing the action to a degree of less than 50% (two points) and (c) completing the action fully (four points), signifying that the highest possible SFBBS score is 28 points. In previous research involving clinical tests with the SFBBS, an SFBBS score lower than 23 meant that the subject was considered to have impaired balance [10,11].

#### 2.3.2. TUG

**Figure 2:**
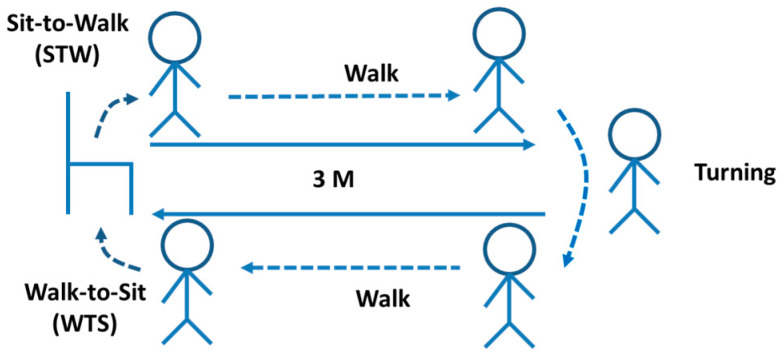
Phases of 3MTUG test.

**Figure 3:**
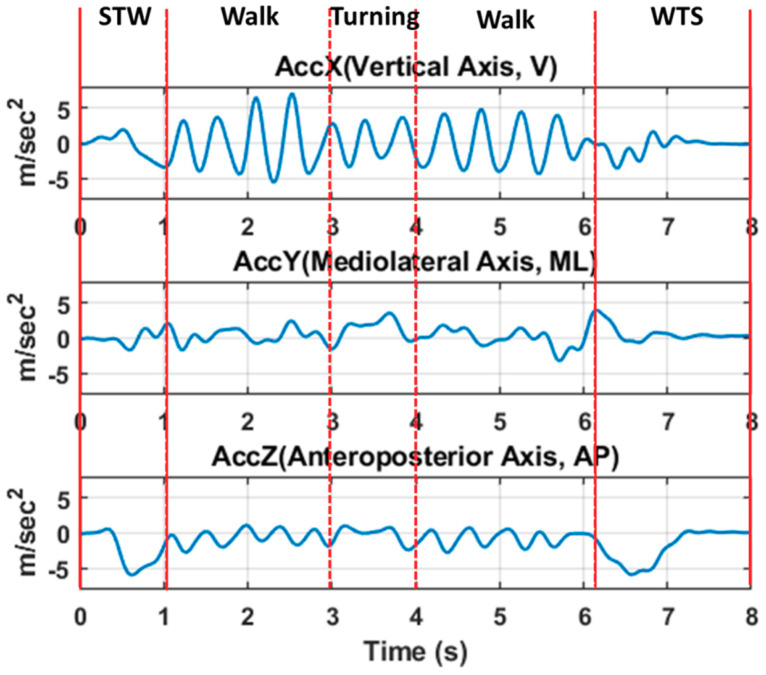
Examples of acceleration signals obtained from a sensor during 3MTUG test (sixth-order Butterworth filter, low-pass filtered at 3 Hz), including four segments (Sit-to-Walk (STW), Walk, Turning, and Walk-to-Sit (WTS)).

After the SFBBS assessment, each subject was asked to perform the 3MTUG test. The entire test was divided into four segments [19]: (i) STW, which involves moving from sitting to standing, followed by gait initiation; (ii) Walk, which involves walking forward and backward for three metres in accordance with usual habits; (iii) Turning, which involves turning back upon arriving at the 3-m target line; and (iv) WTS, which involves turning, followed by moving from standing to sitting (see Figure 2). We referred to a previous study for conducting this experiment. An observer marked the start and end times, and the times to reach the standing position, reach the three-metre mark, turn around, reach the chair, and return to a seated position [23]. The postural stability of each subject was observed during the experiment, and the triaxial acceleration signals of each of the four segments were recorded. These signals were comprised of the triaxial acceleration data collected from the sensor, which were filtered using a sixth-order Butterworth filter and a low-pass filter with 3 Hz frequency suggested in the biomechanics textbook (Winter, 2009). Four segments of acceleration signals after filtering during 3MTUG are shown below (see Figure 3). To better understand the details of the SFBBS evaluation criteria for fall risk when performing the entire 3MTUG and its various phases, we proposed a segment-based evaluation method. We characterised the TUG phases by domain knowledge about the four activities (segments), such as STW, Walk, Turning, and WTS. The more features that can be obtained to conduct MSE analysis for sTUG, the more fall-risk evaluation details that can be investigated and discussed.

### 2.4. Data Analysis

We used MATLAB to conduct signal pre-processing, compute MSE features, construct the regression model, and perform statistical analysis. The complexity of the overall TUG and sTUG for each subject was obtained by MSE analysis. The statistical analysis included multivariable logistic regression, ROC, and AUC, which were used to assess not only the relationship between complexity and postural stability, but also the performance metrics of the fall-risk regression model.

#### 2.4.1. MSE Analysis

**Figure 4:**
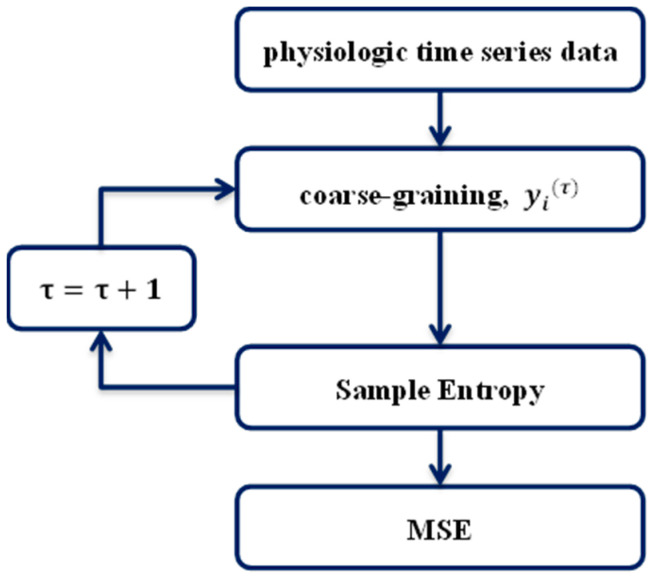
Flow chart of MSE algorithm.

MSE analysis has potentially important applications for evaluating both dynamic models of biological control systems, and bedside diagnostics [12]. It is a method for measuring the complexity of finite-length time series with different scale factors. It is used to analyse the similarity of sequence data, and is currently used widely to analyse physiological data sets [13]. A flow chart of the MSE algorithm is shown in Figure 4.

In calculating the sample entropy for each coarse-grained series, the MSE calculation process involves the following sub-processes [13].

##### 1. Coarse-Graining

**Figure 5:**
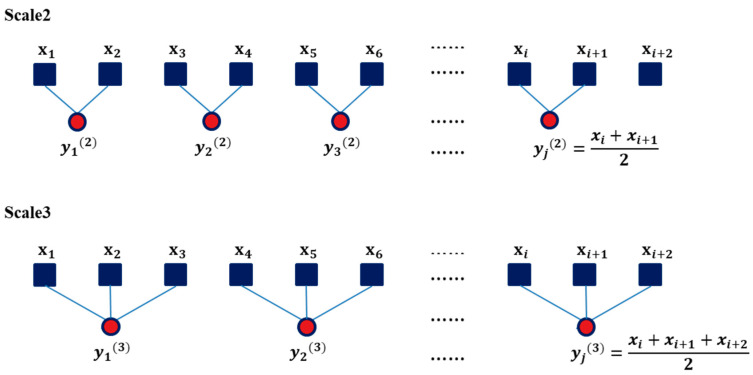
Schematic of coarse-grained procedure.

The “coarse-graining” process is based on the scale factor of the segment windows, calculating the average of the data points for each segment window and forming a new time series, called the coarse-grained time series (see Figure 5). Each element of the new coarse-grained time series, $y_{j}{}^{\left(\tau \right)}$, is calculated according to

yj(τ)=1τ∑i=(j−1)τ + 1jτxi, 1≤j≤Nτ,

where *τ* is the scale factor, *N* is the size of the original dataset, and $x_{i}$ is a data point in the original time series. For a scale factor of 1, the coarse-grained time series is simply the original time series; a scale factor of 2 involves the average of two points in the original time series, which becomes a time series of a scale factor of 2, and so on. The length of each coarse-grained time series is *N*/*τ*. In this study, the scale factor was set from 1 to 6.

##### 2. Sample Entropy

The sample entropy (SampEn) is calculated for each coarse-grained time series, to obtain entropy measures at different scales [15,24]. SampEn at each time scale *τ* is expressed as the negative of the natural logarithm of the conditional probability C(r), where two sequences are to be similar within a tolerance r for m and m+1 consecutive points. The formula is

$$\mathrm{SampEn}\left(N,m,r\right)=-\ln\frac{C^{m+1\;}\left(r\right)}{C^{m}\left(r\right)},$$

where *N* represents data points, *m* indicates the number of consecutive data points, and r is the tolerance for accepting match, which is chosen to be between 10% and 20% of the sample standard deviation of the time series. In this study, the parameters were set as m=2 and r=0.15σ.

##### 3. CI

It is sometimes difficult to compare the heights of multiple curves or determine whether there is a significant difference among them when the curves cross each other. In such cases, CI can be used to evaluate the fall behaviour [9], which is defined as the area under the MSE curve, as proposed by Costa et al. [25]. It can be used to quantify the information expressed by physiological dynamics over multiple scales, and is expressed as

$$\sum_{\tau =1}^{n}\mathrm{SampEn}\left(\tau \right),,$$

where τ is the scale factor. In this study, we used CI to check the significant difference in the overall TUG and sTUG, at n=6.

#### 2.4.2. Adaptive Resampling Procedure in MSE

The data length must be considered when performing MSE analysis; typically, the time series of the length used herein with 1800 data points can be coarse-grained up to scale 6, in which case, the shortest coarse-grained time series should be constituted of 300 data points [25]. Because of sTUG, the segments may not be long enough to calculate the MSE. The literature on the adaptive resampling procedure for MSE shows that the interpolation of adaptive resampling allows the analysis of more scales in a short sequence [26]. In this study, we used the above-described method to provide an evenly spaced time series, which can provide up to 1800 data points for each segment-based TUG, for MSE calculations. During the short sequences of sTUG testing based on the resampling process, we can show the detailed variation in the CI values using MSE analysis, which could help evaluate the fall risk of elderly in the community.

#### 2.4.3. Statistical Analysis

In this study, demographic data and CI values of Table 1 and Table 2 are represented as means ± standard deviations. The CI values were taken as features used as inputs for multivariable logistic regression models to test whether the features could classify a subject as being at risk of falling, considering the SFBBS criterion as the gold standard. The multivariable logistic regression analysis models were used with a favourable outcome (SFBBS < 23) as the dependent variables. The independent variables in the analysis were CI values of a triaxial accelerometer in overall/segment-based TUG. In addition, ROC, which is well-developed in the field of medicine [27], was created to explore the ability of clinical measures and CI values to predict a favourable fall-risk outcome. An AUC of 0.5 indicated no discrimination, that of 1.0 indicated perfect discrimination, and one between 0.7 and 0.9 indicated acceptable or excellent discrimination. The clinical test, according to the elderly with fall risks and those without fall risks, had functional outcomes compared using Student’s *t*-tests (the statistical analysis was deemed significant if p≤0.05).

## 3. Results and Discussion

A subject was considered at risk of falling if her/his SFBBS score was less than 23 points and not at risk of falling if the score was greater than 23 points. The demographic data of subjects at risk of falling (*n* = 19) and not at risk of falling (*n* = 66) are listed in Table 1.

We then used MSE analysis and calculated CI, which checks for significant features related to a favourable fall-risk outcome and obtained multivariable logistic regression models. Using these features of overall TUG and sTUG, the model and performance metric of classification were methodically examined. The results and discussion are divided into four parts: (i) analysis of MSE curves for overall TUG; (ii) analysis of MSE curves for sTUG; (iii) features related to a favourable fall-risk outcome with overall TUG and sTUG; and (iv) analysis of performance metrics for overall TUG and sTUG.

### 3.1. Analysis of MSE Curves for Overall TUG

**Figure 6:**
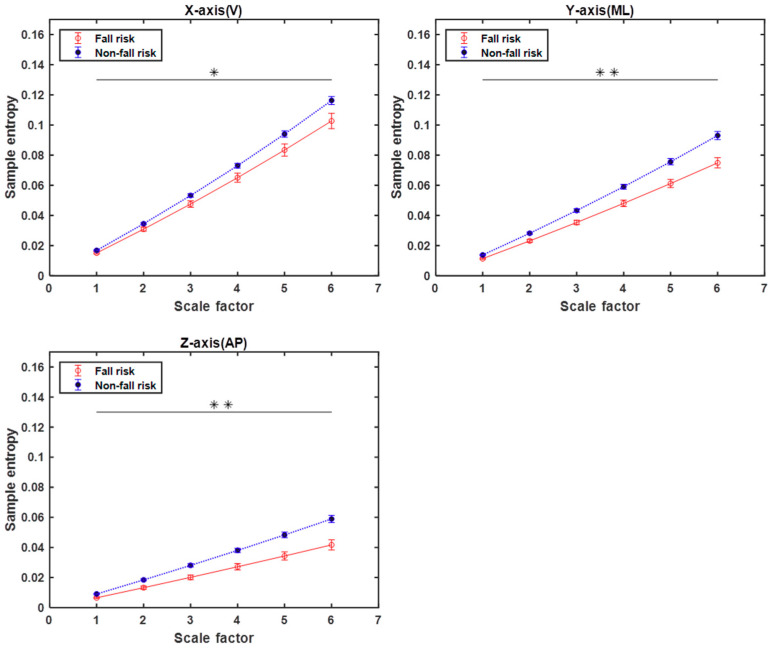
Triaxial MSE curves for overall TUG. Multiscale entropy (MSE) curves of entire activity data of TUG derived from elderly subjects at risk of falling and not at risk for scale factors ranging from 1 to 6. Values are presented as means ± standard errors. The horizontal black lines and asterisks indicate the scales with significant differences in MSE between fall-risk and not-at-risk subjects, where * denotes *p* < 0.05 and ** denotes *p* < 0.005 based on the independent-sample *t*-test.

We plotted the entropy values as a function of scale and analysed the profiles of the triaxial MSE curves obtained, as shown in Figure 6. The triaxial MSE curves of the overall TUG can clearly distinguish between the fall-risk and not-at-risk groups because of the clear separation at larger scale factors; the MSE values are, therefore, important features (*p* < 0.05), which is consistent with a previous study [11]. Moreover, a higher value of entropy was assigned to a time series from the not-at-risk group as compared to the fall-risk group. These results were consistent with those of previous studies reporting an association between higher entropy values and better health [12,13,14].

### 3.2. Analysis of MSE Curves for sTUG

**Figure 7:**
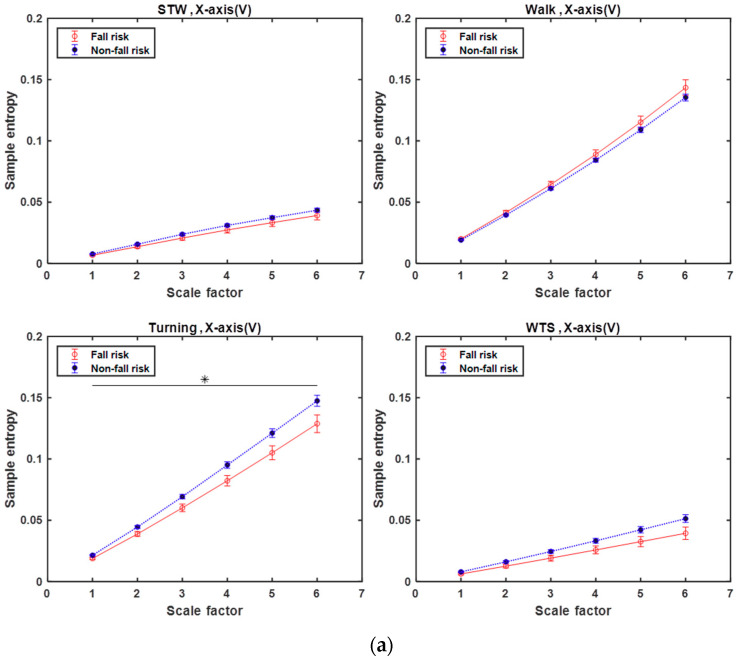

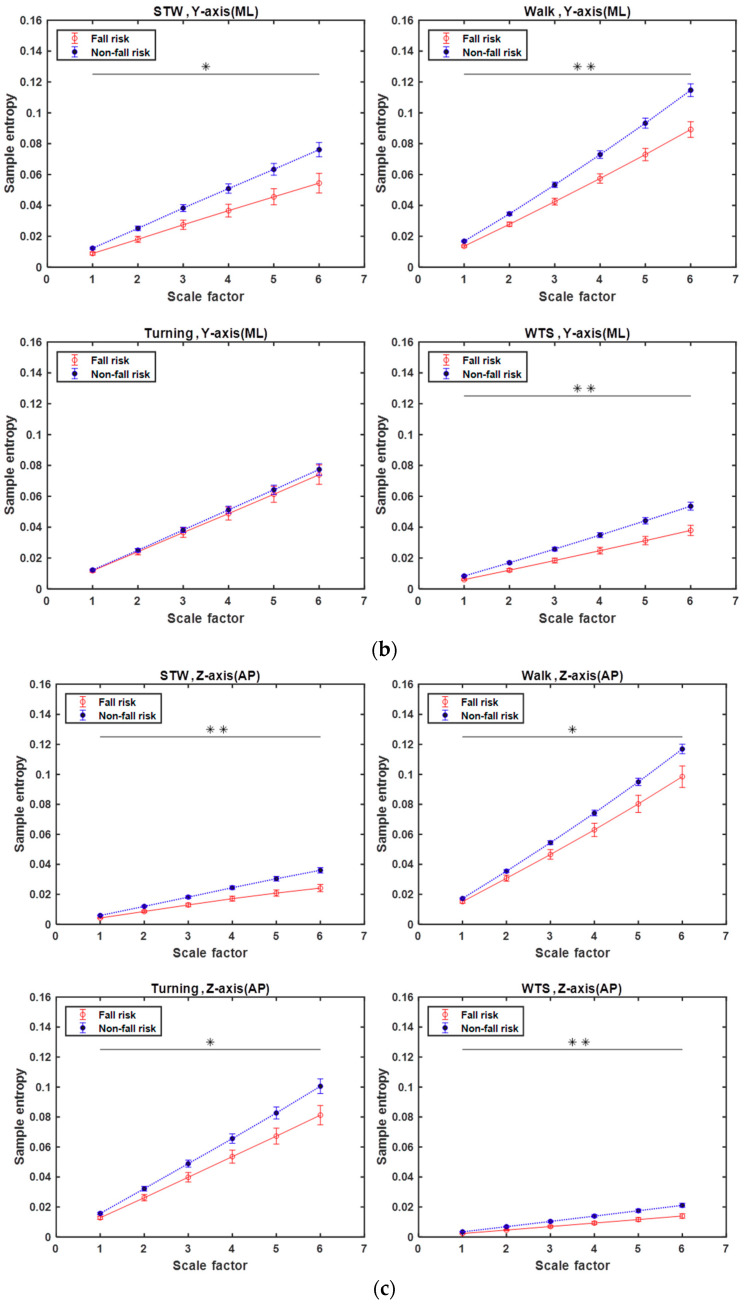
MSE curves derived from the analysis of STW, Walk, Turning, and WTS segments of the accelerometer recordings of fall-risk (red) and not-at-risk (blue) participants along the following projections: (**a**) vertical, *X*-axis; (**b**) mediolateral, *Y*-axis; and (**c**) anterior–posterior, *Z*-axis. The values are presented as means ± standard errors. The horizontal black lines and asterisks indicate the scales with significant differences in MSE between fall risk and not-at-risk subjects, where * is *p* < 0.05 and ** is *p* < 0.005 by independent-sample *t*-tests.

From Figure 7a–c, the triaxial MSE curves of segment-based activity are described as follows.

- For the *X*-axis (V) shown in Figure 7a, the MSE curves of the Turning segment clearly not only have higher entropy for the not-at-risk subjects but also offer better classification performance (*p* < 0.05) compared to the other segments. This is consistent with a previous study [28], which noted that turn-based features are important predictors because they contain useful biomechanical information that can improve the prospective fall-risk classification for healthy elderly people. In the other segments, the MSE curves show a nonsignificant difference between the two groups.
- For the *Y*-axis (ML) shown in Figure 7b, except for the Turning segment, the other three segments show a significant distinction (*p* < 0.05) between the two groups. In the Walk segments, the MSE curves show the gait complexity of swaying left and right, which is supposedly related to the arms swing when walking. Because of the walking duration, an arm swing is associated with postural stability [29] and can enhance gait stability [30]. Therefore, the MSE curve of the not-at-risk among the elderly is higher. In the STW and WTS segments, the MSE curves showed that the elderly subjects at risk of falling have lower complexity. Weiss et al. indicated that the ability to perform the STW and WTS subtasks are largely dependent on lower extremity strength [18]. Furthermore, Hausdorff et al. mentioned that the lower extremity function was significantly different among the fallers than among the non-fallers [31]. Thus, we can speculate that the low dynamic complexity of STW/WTS subtasks is associated with weaker, lower extremities.
- For the *Z*-axis (AP) shown in Figure 7c, the Walk and Turning segments show a significant difference (*p* < 0.05) between the two groups, and the STW and WTS segments show a stronger significant difference (*p* < 0.005). Besides, when referring to the transition subtask involving standing up or sitting down, the body must bend in forward–backward displacement, and the AP-axis is seemingly an important axis, especially in the STW and WTS segments, a result that is similar to that obtained in a previous study [11], which mentioned that the extracted features of the transition subtask in the AP-axis are significant.

In summary, the turning segments of the *X*-axis (V); the STW, WTS, and Walk segments of the *Y*-axis (ML); and all segments of the *Z*-axis (AP), seem to exhibit quite a discriminating power during sTUG. Costa et al. [32] asserted that complexity is associated with ‘meaningful structural richness’ incorporating correlations over multiple spatiotemporal scales. Their study result showed that the MSE values of the Walk/Turning segment are higher than those of the STW/WTS segment. Thus, we could infer that Walk and Turning segments contain more meaningful physiological information about postural stability than STW and WTS segments.

### 3.3. Features Related to Favourable Fall Risk Outcome with Overall TUG and sTUG

The complexity of the dynamics for both the overall TUG period and individual TUG segments was significantly higher for subjects in the not-at-risk than those in the fall risk (see Table 2). The four exceptions included STW, Walk and WTS along the vertical direction, and Turning along the mediolateral direction.

From the overall TUG analysis in Table 2, all triaxial CI values can distinguish a significant (*p* < 0.05) difference between the fall risk and not-at-risk groups, making them important features, as is consistent with a previous study [11]. For sTUG, there are significant predictors of fall risk for eight of the 12 CI features, which may offer improvements for early fall-risk assessment. It is reasonable to employ MSE analysis and use these significant features to construct the regression model for assessing fall risk. Moreover, we found higher CI values for the not-at-risk group compared to the fall risk group, while previous studies have associated higher complexity with better health [12,13,14].

The larger CI values indicate that the trunk triaxial acceleration assessed by the wearable sensor recordings is more irregular and complex for the not-at-risk subjects compared to the fall risk ones. Because lower SampEn values were assigned to more regular time series, while higher SampEn values were assigned to more irregular and less predictable time series [13], we could infer that in the Walk segment, significant features, including Walk(ML) and Walk(AP), reflected an increase in the irregular variations and complexities of step width and walking acceleration with not-at-risk subjects. In the STW and WTS segments, they showed an increase in the complexity of variations of acceleration for the bending in the AP direction (forward–backward) and ML direction (right–left) of the not-at-risk subjects. Elderly people who are not at risk of falling have larger CI values in the Turning segment and exhibit significant irregular variations in acceleration in the V and AP directions, a similar result to that obtained in a previous study [28], which noted that Turning-based features have been found to be important predictors for assessing fall risk because of the decreased stability when elderly people navigate turns.

### 3.4. Analysis of Performance Metrics for Overall TUG and sTUG

**Figure 8:**
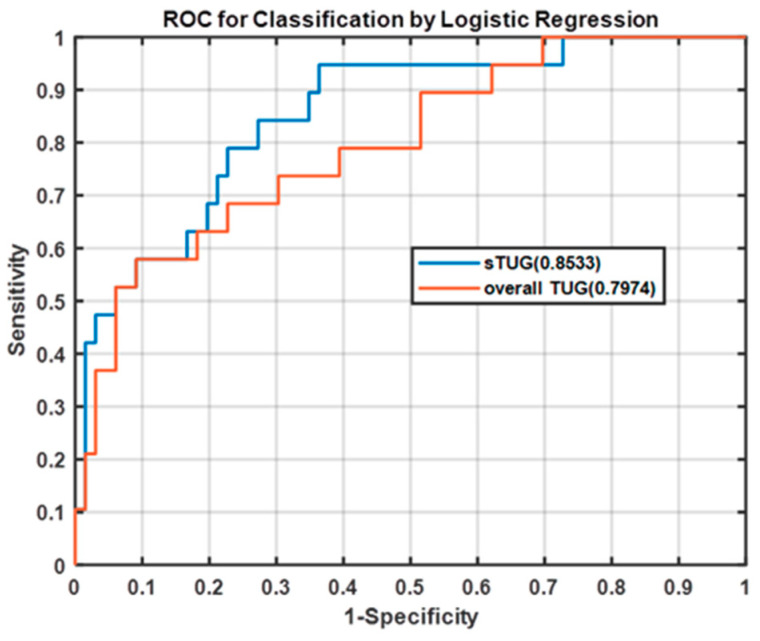
Receiver operating characteristic (ROC) curves for overall TUG and sTUG prediction model.

Through the logistical regression analysis with significant features, the performance of classification, such as AUC, can be obtained, which was compared and taken as a performance metric of the regression model in overall TUG and sTUG (see Figure 8).

We used the AUC for predicting a favourable outcome for elderly people with fall risk in the overall TUG and sTUG model. For overall TUG, the logistical regression model was built from the triaxial features of the CI values. Its AUC range was 0.797 (95% CI 0.683 to 0.912), meaning that the discrimination of the regression model was acceptable. Similarly, for sTUG, the significant features associated with fall risk, including STW (ML), STW (AP), Walk (ML), Walk (AP), Turning (V), Turning (AP), WTS (ML), and WTS(AP), were selected to construct the logistic regression model. Its AUC was improved, being 0.853 (95% CI 0.759 to 0.948). Thus, the complexity analysis of sTUG can reveal more information about gait and postural stability evaluations for fall risk.

## 4. Conclusions

The triaxial acceleration signals of the overall TUG and sTUG were more irregular and complex for the not-at-risk subjects compared to the fall risk subjects. These results indicate that, compared to overall TUG, sTUG can reveal more information about postural (transition/gait) stability from MSE analysis and provide more predictors of fall-risk for assessment among community-dwelling elderly people. In the future, we plan to investigate how the discriminatory power of MSE compares with that of other entropy measures in this database, such as composite multiscale entropy and multiscale permutation entropy.

## Figures and Tables

**Table 1:** Demographic data of subjects at risk of falling and those not at risk of falling.

	Fall Risk (n = 19)	Non-Fall Risk (n = 66)
Age	78.37 ± 7.54	75.47 ± 6.74
Gender		
male	3	15
female	16	51

**Table 2:** CI features of overall TUG and sTUG for subjects.

	Fall Risk (n = 19)	Non-Fall Risk (n = 66)	*p* Value
Overall TUG			
V	0.345 ± 0.071	0.388 ± 0.069	0.0266 *
ML	0.254 ± 0.049	0.313 ± 0.070	0.0001 **
AP	0.143 ± 0.050	0.201 ± 0.064	0.0002 **
sTUG			
STW(V)	0.141 ± 0.056	0.159 ± 0.049	0.2125
STW(ML)	0.191 ± 0.094	0.266 ± 0.127	0.0074 *
STW(AP)	0.088 ± 0.037	0.127 ± 0.051	0.0007 **
Walk(V)	0.474 ± 0.074	0.449 ± 0.066	0.1922
Walk(ML)	0.304 ± 0.067	0.386 ± 0.101	0.0002 **
Walk(AP)	0.334 ± 0.094	0.393 ± 0.071	0.0184 *
Turning(V)	0.434 ± 0.100	0.499 ± 0.115	0.0217 *
Turning(ML)	0.257 ± 0.094	0.268 ± 0.100	0.6385
Turning(AP)	0.281 ± 0.097	0.346 ± 0.134	0.0245 *
WTS(V)	0.136 ± 0.075	0.175 ± 0.086	0.0592
WTS(ML)	0.131 ± 0.049	0.184 ± 0.070	0.0005 **
WTS(AP)	0.049 ± 0.023	0.073 ± 0.039	0.0012 **

* indicate *p* < 0.05, ** indicate *p* < 0.005 between two groups.
